# PRESIDENTIAL SYMPOSIUM: IMPACT OF DISCRIMINATORY POLICIES ON LGBTQ+ OLDER ADULTS’ HEALTH AND WELL-BEING AND HOW TO COMBAT THEM

**DOI:** 10.1093/geroni/igad104.1360

**Published:** 2023-12-21

**Authors:** Jung Kwak, Karen Fredriksen-Goldsen

## Abstract

Despite some advances in LGBTQ+ rights over the past few decades, many states lack legal protections against discrimination based on sexual orientation or gender identity, and the current political climate raises concerns about future challenges to LGBTQ+ rights. This symposium explores the implications of discriminatory policies on the health, housing, and economic well-being of LGBTQ+ older adults, and solutions for promoting empowerment, equity, and inclusion throughout the care continuum, from long-term services and supports to end-of-life care. The first presentation describes a complex paradox in which sexual and gender minority (SGM) older adults avoid formal services due to fear of discrimination and poor treatment despite significant health disparities and a lack of informal support, discusses opportunities to improve access and health through education and awareness, and elucidates implications for policy and research. The second presentation describes geographic variations in access to, and receipt of gender-affirming surgery among transgender and gender-diverse Medicare beneficiaries using national Medicare data and discusses policy implications. The third paper focuses the role of spirituality and faith in the lives of LGBTQ+ older adults and reports findings from a mixed-methods study on how involvement in faith communities shape their end-of-life care perceptions and preparation. The fourth presentation describes barriers and facilitators to communicating with SGM hospice patients and caregivers about their sexual orientation and gender identity and recommendations for developing communication training to promote authentic end-of-life care for this population. Karen Frederickson-Goldsen as discussant will provide policy and practice implications of these important works.

